# A Causal Role for Gastric Rhythm in Human Disgust Avoidance

**DOI:** 10.1016/j.cub.2020.10.087

**Published:** 2021-02-08

**Authors:** Camilla L. Nord, Edwin S. Dalmaijer, Thomas Armstrong, Kate Baker, Tim Dalgleish

**Affiliations:** 1 Medical Research Council Cognition and Brain Sciences Unit, University of Cambridge, 15 Chaucer Road, Cambridge CB2 7EF, UK; 2 Department of Psychology, Whitman College, 345 Boyer Avenue, Walla Walla, WA 99362, USA; 3 Cambridgeshire and Peterborough NHS Foundation Trust

**Keywords:** disgust, avoidance, visceral, interoception, nausea, eye tracking, gut-brain interaction, gastric, somatic, emotion

## Abstract

Rotten food, maggots, bodily waste—all elicit disgust in humans. Disgust promotes survival by encouraging avoidance of disease vectors^1^ but is also implicated in prejudice toward minority groups; avoidance of environmentally beneficial foods, such as insect protein; and maladaptive avoidance behavior in neuropsychiatric conditions.^2^, ^3^, ^4^, ^5^ Unlike fear, pathological disgust is not improved substantially by exposure therapy clinically,^6^ nor in experimental work does behavioral avoidance of disgusting images habituate following prolonged exposure.^7^^,^^8^ Under normal physiological conditions, perception of disgusting stimuli disrupts myoelectrical rhythms in the stomach,^9^, ^10^, ^11^, ^12^, ^13^ inducing gastric dysrhythmias that correlate with neural signatures of disgust.^11^ However, the causal role of gastric rhythm in disgust avoidance is unknown. We manipulated gastric rhythm using domperidone, a peripheral dopamine D2/D3 antagonist and common anti-emetic, at a dose (10 mg) that acts to convert gastric dysrhythmias to normal rhythms.^9^ In a preregistered, randomized, double-blind, placebo-controlled crossover design in 25 healthy volunteers (aged 18–25), we measured the effects of domperidone on core disgust avoidance, using eye tracking to measure implicit (oculomotor) avoidance of disgusting images (feces) before and after an “exposure” intervention (monetary reinforcement for looking at disgusting images).^7^^,^^8^ We find that domperidone significantly reduces oculomotor disgust avoidance following incentivized exposure. This suggests that domperidone may weaken the “immunity” of disgust to habituation, putatively by reducing gastric dysrhythmias during incentivized engagement with disgusting stimuli. This indicates a causal role for disgust-related visceral changes in disgust avoidance, supporting the hypothesis that physiological homeostasis contributes to emotional experience.

## Highlights

• Healthy volunteers were given domperidone to normalize gastric rhythm or placebo
• We measured the effect of an exposure paradigm on oculomotor disgust avoidance
• Domperidone reduced oculomotor disgust avoidance after the exposure intervention
• This suggests gastric rhythm has a causal role in disgust avoidance

## Teaser

It is unknown whether gastric dysrhythmias contribute to disgust avoidance. By normalizing gastric state with domperidone and measuring oculomotor avoidance of disgusting images, Nord et al. discover that domperidone reduces oculomotor disgust avoidance following an exposure task. This suggests that gastric rhythm is one cause of disgust avoidance.

## Results

**Figure 1:**
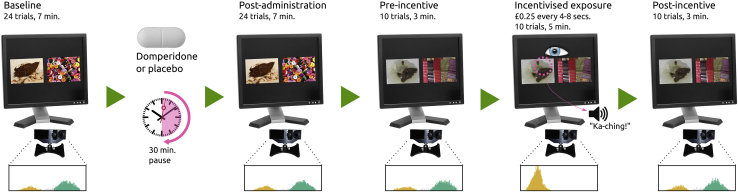
Experimental Procedure (Disgust Task) Participants visited twice, for domperidone and placebo administration, at least 7 days apart. At each session, we collected eye-tracking data over 24 trials (7 min) at baseline, 30 min after domperidone/placebo administration, and before and after incentivized exposure, when participants were reimbursed for ocular engagement with the disgusting stimulus. Histograms show horizontal eye gaze fixation positions (yellow histography on left indicates the disgusting stimulus; position was randomized in the experiment), weighted by fixation duration. Note that the original stimuli are not licensed for publication; these images are provided for illustration. Control (reward task) administration occurred after baseline measurement and again after post-incentive measurement (not pictured).

Our preregistered outcome was the effect of domperidone on oculomotor disgust avoidance, quantified as the proportional difference between gaze dwell times on disgusting (feces) versus neutral (scarves/buttons) images. We tracked gaze at baseline and post-drug/placebo administration (pre-exposure phase) and pre- and post-gaze-contingent, reward-incentivized exposure procedure with novel images (exposure phase; Figure 1).^7^ We predicted that attenuation of gastric dysrhythmias by domperidone, when paired with incentivized exposure, would reduce oculomotor disgust avoidance.

We also measured the effects of domperidone on reinforcement learning, which is worsened by central D2/D3 receptor antagonists.^14^, ^15^, ^16^, ^17^ Given the extensive evidence against central dopaminergic effects of domperidone, we predicted a null effect of domperidone on reinforcement learning.

### Pre-exposure Phase

A linear mixed-model (LMM) of dwell-time proportions confirmed a significant disgust avoidance effect pre-exposure (β_stimulus_ = −1.32; confidence interval [CI] = [−1.41, −1.23]; *Z* = −28.56; p < 0.001), increasing over trials (β_stimulus^∗^trial_ = −0.14; CI = [−0.23, −0.05]; *Z* = −3.03; p = 0.002).

**Figure 2:**
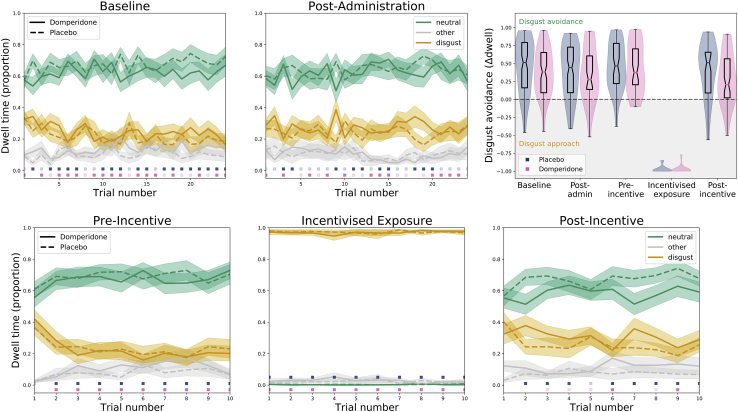
Average Dwell Time Proportions for the Disgusting (Yellow) and Neutral (Green) Stimuli and Non-stimulus Fixations (Gray) Shaded areas indicate the within-participant standard error of the mean. Colored boxes above the x axis indicate a significant difference in dwell time proportion between the disgusting and neutral stimuli time for each medication condition (blue, placebo; pink, domperidone; transparent for uncorrected α = 0.05; opaque after for Holm-Bonferroni correction). The subplots correspond to the task phases and histograms in Figure 1. The top-right panel shows the distribution of proportional dwell-time differences (averaged across trials within each block) for placebo (blue) and domperidone (pink). Notches in the boxplots indicate the within-participant 95% confidence interval around the median.

Despite randomization, pre-drug administration, participants showed lower levels of disgust avoidance when allocated to receive domperidone compared to when allocated to placebo (β_stimulus^∗^drug_ = 0.23; CI = [0.10, 0.36]; *Z* = 3.50; p < 0.001); this effect did not differ pre- to post-drug administration (β_stimulus^∗^drug^∗^phase_ = −0.10; CI = [−0.28, 0.08]; *Z* = −1.05; p = 0.294). There was no differential effect of medication on disgust avoidance from baseline to post-administration (Figure 2, top row) prior to the incentivized exposure period, although avoidance reduced overall (β_phase_ = 0.10, CI = [−0.19, −0.01], *Z* = −2.13, p = 0.033; β_stimulus^∗^phase_ = 0.17, CI = [0.05, 0.30], *Z* = 2.67, p = 0.008).

No other pre-exposure main or interaction effects were significant (p > 0.058). The full model incorporated 4,744 observations from 25 participants (observations per participant min = 170; max = 192; mean = 189.8); post hoc models incorporated 1,186 observations (observations per participant: pre-administration: min = 42, max = 48, mean = 47.4; post-administration: min = 39, max = 48, mean = 47.4). Both the evidence ratio and the Bayes factor for the model tended toward infinity, providing conclusive evidence against the null model. The reported baseline offset was reflected in post hoc LMMs that showed a supposed drug effect *before* (β_drug_ = −0.15; CI = [−1.24, −0.06]; *Z* = −3.39; p = 0.001; ER_1_ = 8.96; BF_10_ = 1.41), but not after drug/placebo administration (β_drug_ = −0.08, CI = [−1.17, 0.01]; *Z* = −1.83; p = 0.068; ER_0_ = 4.52; BF_01_ = 57.31).

### Effects of Incentivized Exposure

Before and after incentivized exposure, participants exhibited significant oculomotor disgust avoidance (β_stimulus_ = −1.30; CI = [−1.44, −1.16]; *Z* = −18.13; p < 0.001).

In support of our preregistered hypothesis, there was a significantly greater reduction in disgust avoidance from pre- to post-exposure following administration of domperidone (β_stimulus^∗^drug^∗^phase_ = 0.35; CI = [0.07, 0.63]; *Z* = 2.43; p = 0.015; Figure 2, bottom row), confirmed by a post hoc LMM of neutral-disgust dwell differences between domperidone and placebo (β = −0.28; CI = [−0.42, −0.13]; *Z* = −3.67; p < 0.001).

No other main or interaction effects were significant (p > 0.054). The full model incorporated 1,972 observations from 25 participants (observations per participant min = 64; max = 80; mean = 78.9); post hoc models incorporated 492, 500, and 494 observations, respectively (observations per participant pre-incentive: min = 15, max = 20, mean = 19.8; during incentive: min = 20, max = 20, mean = 20; post-incentive: min = 17, max = 20, mean = 19.7). The evidence ratio (ER_1_ = 4.75e163) and the Bayes factor (BF_10_ = 3.02e145) for the model provided conclusive evidence against the null model.

This was corroborated by post hoc tests showing evidence for the null model over LMMs of the main effect of drug on the difference in oculomotor disgust avoidance pre-incentive (β_drug_ = −0.03; CI = [−1.18, 0.12]; *Z* = −0.43; p = 0.667; ER_0_ = 13.14; BF_01_ = 107.46) and during incentivized exposure (β_drug_ = 0.02; CI = [−1.13, 0.17]; *Z* = 0.23; p = 0.816; ER_0_ = 14.02; BF_01_ = 115.35) but evidence for a drug effect post-incentive (β_drug_ = −0.24; CI = [−0.39, −0.09]; *Z* = −3.17; p = 0.002; ER_1_ = 10.10; BF_10_ = 1.24). Although the Bayesian information criterion (BIC)-based Bayes factor remained inconclusive, the Akaike information criterion (AIC)-based evidence ratio suggested a drug effect was 10 times more likely than the null hypothesis, and this was supported by frequentist statistics (p = 0.002).

### Self-Reported Disgust

Self-reported disgust, measured before and after administration (“pre-exposure phase”) and before and after incentivized exposure (“exposure phase”), was higher for disgusting compared to neutral stimuli for both pre-exposure (β_stimulus_ = 1.83; CI = [1.56, 2.10]; *Z* = 13.31; p < 0.001) and exposure phases (β_stimulus_ = 2.01; CI = [1.79, 2.23]; *Z* = 17.88; p < 0.001). For dwell time, interaction effects of stimulus and drug for pre-exposure (β_stimulus^∗^drug_ = −0.39; CI = [−0.77, −0.01]; *Z* = −2.01; p = 0.045) and exposure (β_stimulus^∗^drug_ = −0.37; CI = [−0.68, −0.06]; *Z* = −2.31; p = 0.021) reflected baseline offsets between the placebo and domperidone visits. No other main or interaction effects were significant (p > 0.064). Crucially, there was no interaction of stimulus, drug, and phase pre-exposure (β_stimulus^∗^drug^∗^phase_ = 0.19; CI = [−0.35, 0.73]; *Z* = 0.68; p = 0.498) or during exposure (β_stimulus^∗^drug^∗^phase_ = 0.22; CI = [−0.22, 0.66]; *Z* = 0.99; p = 0.322).

This suggests that, unlike oculomotor disgust avoidance, self-reported disgust was not affected by domperidone (no missing data; conclusive evidence for LMMs compared to their null models [pre-exposure phase: ER_1_ = 3.78e50, BF_10_ = 3.67e45; exposure phase: ER_1_ = 1.73e65, BF_10_ = 1.67e60]).

### Control Task

Performance on a reinforcement learning task was unaffected by domperidone. This was true for the proportion of won trials but also for parameters derived through computational modeling: learning rate (η) and inverse choice temperature (β) were unaffected, even when learning rate was computed separately for wins and non-wins (Table 1). In fact, the null model was 14–107 (ER_0_ based on AIC) or 680–5,328 times (BF_01_ based on BIC) times more likely to explain the data than linear mixed effects models that tested main effects of drug and phase and their interaction. Post hoc linear mixed effects models testing the main effect of drug on parameter differences between the pre- and post-administration phases showed consistent evidence for the null model (ER_0_ between 1.37 and 4.50 and BF_01_ between 3.56 and 11.71). There were no missing data.

**Table 1:** Effect of Domperidone on Reinforcement Learning Task

	Drug		Phase		Drug^∗^Phase		Evidence for Null Model		Evidence against Drug	
	β	p	β	p	β	p	ER_0_	BF_01_	ER_0_	BF_01_
P(win)	0.366	0.131	0.213	0.379	−0.442	0.197	38.60	1,921.70	1.37	3.56
η	0.190	0.366	0.138	0.510	−0.118	0.692	107.02	5,327.99	3.61	9.39
β	0.202	0.398	0.216	0.366	−0.017	0.960	32.42	1,614.17	4.16	10.82
η_win_	0.380	0.150	0.189	0.473	−0.085	0.821	13.66	680.07	4.07	10.59
η_lose_	0.204	0.355	0.068	0.759	0.013	0.968	60.62	3,018.00	4.21	10.96
β_win/lose_	−0.003	0.992	0.094	0.729	0.016	0.967	80.07	3,986.27	4.50	11.71

Results of linear mixed effects testing the main effects of the day of domperidone or placebo administration, phase (pre- versus post-administration), and their interaction (standardized coefficient β and associated p values reported); evidence against the full mixed effects (compared against a null model of only an intercept); and evidence against a specific drug effect from a separate linear mixed effects model to test only the main effect of drug on the parameter difference between post- and pre-administration phases (under “Evidence against Drug”). The evidence ratio for the null model (ER_0_) was computed from the difference between Akaike information criterions (AICs) for each model and its null model, and Bayes factors for the null (BF_01_) were computed from the difference between Bayesian information criterions (BICs), with values over 1 indicating evidence for the null model.

## Discussion

We tested whether incentivized exposure to disgusting images during a normalized gastric state (after domperidone administration) reduces subsequent oculomotor disgust avoidance. We found that domperidone reduced oculomotor disgust avoidance following incentivized exposure to disgusting stimuli. We found no evidence for domperidone-induced changes in subjective disgust or disgust avoidance before incentivized exposure. This suggests that normalizing gastric state specifically during motivated engagement with disgusting stimuli results in reduced disgust avoidance that endures beyond the exposure phase. This is particularly striking given the “immunity” of oculomotor disgust avoidance to repeated and incentivized exposure under placebo.^7^ This indicates a causal role for gastric state in exposure-induced oculomotor disgust avoidance.

Our study was motivated by previous evidence that core disgust images reduce normogastric activity,^11^^,^^12^ although domperidone restores normogastric activity.^18^ We predicted that gastric dysrhythmias may be one important cause of disgust avoidance. We report that domperidone reduces oculomotor avoidance of disgusting images after an exposure intervention. Supporting many previous studies reporting peripheral (rather than central) effects of domperidone, we also report that domperidone does not alter reinforcement learning. Performance on reinforcement learning tasks is worsened by D2/D3 receptor antagonists sulpiride,^15^ pramipexole,^16^^,^^19^ and cabergoline^17^ and enhanced by D2/D3 agonist haloperidol.^17^ Thus, if domperidone were to unexpectedly affect central dopaminergic function, it would likely modulate reinforcement learning. Here, Bayesian statistics showed conclusive evidence against an effect of domperidone on reinforcement learning. Therefore, we infer that the peripheral actions of domperidone—restoration of normogastric activity while viewing disgusting images during the exposure intervention—reduced subsequent oculomotor avoidance of disgusting images. This suggests that gastric dysrhythmias during engagement with core disgust stimuli is one cause of disgust avoidance.

The role of gastric myoelectrical activity in disgust is usually interpreted in the context of somatic theories of emotion: peripheral physiological changes that contribute to the experience of a unique emotional state.^11^ Our data largely align with these theories. By combining an exposure-based intervention (monetary reinforcement for viewing disgusting images) with pharmacological manipulation (domperidone, which restores normal gastric myoelectrical activity),^9^ we demonstrate that normalizing gastric myoelectrical activity with domperidone during disgust exposure significantly reduces post-exposure disgust-related avoidance. Our key outcome, oculomotor avoidance, represents a sensitive objective measure of the primary behavior evoked by disgusting stimuli—avoiding sensory contact.^7^

Oculomotor disgust avoidance does not respond to prolonged or even reinforced exposure,^7^^,^^8^ a robust effect we replicate in our placebo condition. This parallels the often-described clinical intractability of pathological disgust to exposure therapy.^6^ By normalizing a physiological concomitant of disgust during motivated exposure (putatively, abnormal gastric myoelectrical activity), domperidone may weaken this apparent immunity of disgust avoidance to exposure. It is particularly notable that we do not report domperidone-induced changes in oculomotor disgust avoidance before incentivized exposure. This implies that any reduction in disgust avoidance requires motivated engagement with the disgusting stimulus during a normalized gastric state.

Interestingly, despite the effect of domperidone on post-exposure disgust avoidance, we do not find an effect of domperidone on self-reported disgust. Like other emotions, disgust may be composed of dissociable components.^20^^,^^21^ In the case of fear, reductions in avoidance behavior after exposure therapy (for example, avoidance of a spider) are not always accompanied by changes in self-reported fear.^20^^,^^21^ Likewise, domperidone may have altered disgust avoidance (putatively via physiological changes to gastric experience) without changing disgust self-report. Possibly, repeated exposure to disgusting stimuli during domperidone could eventually alter subjective disgust, akin to antidepressant drugs, which evoke immediate effects on emotion perception but require long-term exposure before improving mood.^22^^,^^23^

### Limitations and Future Directions

Our work harnesses eye tracking and pharmacology to provide a link between pharmacological manipulation of gastric activity and disgust-related avoidance, but to fully establish *how* dysregulated gastric activity might drive oculomotor avoidance, future studies require measures of disgust-related neural and gastric myoelectrical activity before and after incentivized exposure.^11^ The actions of domperidone are primarily on dopamine receptors in the upper gastrointestinal tract; antagonism of these receptors alters the myoelectrical rhythms of smooth muscle.^9^^,^^24^ Domperidone has the highest affinity for (and concentration in) gastrointestinal tissue,^24^ but there is some penetrance in neural regions without a blood-brain barrier. In particular, domperidone also evokes anti-emetic effects via dopamine antagonism in the area postrema in the medulla oblongata, which itself can alter gastrointestinal myoelectrical activity.^25^ Therefore, our experiment cannot adjudicate between direct or indirect (or both) effects of domperidone on gastric rhythm.

However, we can be relatively certain that the effect of domperidone on disgust avoidance is unlikely to be driven by dopamine receptors involved in reward, emotion, or other cognitive processes, not only because of the extensive evidence against any actions of domperidone on the central dopaminergic systems in motor, prefrontal, or striatal regions,^25^, ^26^, ^27^ but also because of our empirical demonstration of evidence against an effect on any component of reinforcement learning, known to be altered by central D2/D3 receptor antagonism.^14^, ^15^, ^16^ Although we found no effect of domperidone on our control task, there may still be some circumstances in which reinforcement learning is affected by gastric state. For example, a normalization of gastric state could alter reinforcement learning of food rewards (the most common reinforcement used in animal research) or even instrumental response vigor, which in animals is enhanced by the gastric state of hunger.^28^ Therefore, the effect of domperidone should also be tested on paradigms using food rewards and/or response vigor.^29^

We tested participants in a fasted state to replicate the conditions of previous studies where core disgust images (including of feces) elicited deviations from normogastria.^12^ However, in a fasted state (or in uncomfortable fullness), gastric myoelectrical activity is unstable.^30^ Although fasted state was identical between placebo and domperidone conditions, it is still possible that this specific effect of domperidone on disgust avoidance would not be found under other hunger states. Moreover, the effect of domperidone on disgust avoidance (under fasted or fed conditions) should be measured in populations suffering from pathological disgust symptoms to ascertain its translational potential. In both cases, the specific gastric mechanisms driving our effect (and any mediators) require corroboration through direct testing using electrogastrogram or similar methodologies.

To our knowledge, there have not to date been any experiments measuring avoidance following peripheral dopamine antagonism in the context of other emotional or cognitive tasks. Therefore, it is possible that domperidone instantiates a more generalized anxiolytic effect on avoidance; a broader effect of gastric state on avoidance would certainly have far-reaching implications for neuroscience and psychology. An essential future endeavor will be to establish the effects of domperidone on emotion-specific versus more general avoidance behavior, which must also involve a more comprehensive understanding of the gastric changes associated with emotion experience. We would predict our finding only extends to emotions that (like core disgust) evoke gastric dysrhythmias. In contrast, other types of disgust (such as painful-injury disgust) may be mediated by parasympathetic changes in the cardiovascular system.^11^^,^^12^ Therefore, we would not necessarily predict domperidone to alter oculomotor avoidance of painful-injury disgusting images or other types of disgust mediated by non-gastric changes. However, our findings might extend to other types of core disgust, such as images of vomit or rotten food, given their association with gastric-state changes.^12^ One possibility is that our results extend to types of disgust that involve orogastric reactions (e.g., gagging and nausea), but not those that do not. This has implications for certain types of moral disgust, such as violation of sexual norms, which elicit orogastric rejection, termed “moral dyspepsia”.

### Conclusions

The sight of feces, the smell of rot, and the taste of sour milk each evokes a distinct exterosensory experience but a common emotional experience—disgust—marked by characteristic changes in the brain and viscera.^11^^,^^31^ Here, we find evidence that pharmacologically reducing disgust-related visceral changes decreases oculomotor disgust avoidance following a period of motivated exposure. This supports the contribution of peripheral physiology to disgust-related avoidance behavior and may reveal new routes to treating maladaptive disgust.

## STAR★Methods

### Key Resources Table

**Table:** 

REAGENT or RESOURCE	SOURCE	IDENTIFIER
**Deposited Data**		

Raw and analyzed data	This paper	https://osf.io/yxf5r/

**Experimental Models: Organisms/Strains**		

Humans, recruited from the general population in Cambridge, UK, meeting the inclusion criteria described in STAR Methods	N/A	N/A

**Software and Algorithms**		

Linear mixed effect models	This paper	https://osf.io/yxf5r/
Reward task code and stimuli	This paper	https://osf.io/yxf5r/
Gaze task code and stimuli	This paper	https://osf.io/yxf5r/
Plotting functions from data	This paper	https://osf.io/yxf5r/
MATLAB	Mathworks	RRID: SCR_001622
Python	Python Software Foundation	RRID: SCR_008394

**Other**		

Study preregistration	This paper	https://osf.io/nueps

### Resource Availability

#### Lead contact

Further information and requests for resources should be directed to and will be fulfilled by the Lead Contact, Camilla Nord (camilla.nord@mrc-cbu.cam.ac.uk).

#### Materials Availability

This study did not generate new materials, including unique reagents, plasmids, mouse lines, or any other non-data material.

#### Data and Code Availability

The datasets and code generated during this study are available on the Open Science Framework (OSF) (https://osf.io/yxf5r/). There are no restrictions to the availability of the dataset or code. Original/source data for all figures in the paper are also available on OSF.

### Experimental Model and Subject Details

Datasets from 32 human volunteers were collected. Twenty-seven datasets included both placebo and domperidone sessions. Two participants were excluded: one due to technical issues, and the other because they showed no effect of incentivised exposure, likely due to misunderstanding of instructions (note conclusions from the results did not change whether this participant was included or not). The final dataset included 25 participants, aged 18-35 (mean age: 26.7); 14 identified as female and 18 as male. Participants were healthy and free of medical contraindications to domperidone (including any current medication except oral contraceptives, and any gastrointestinal, renal, hepatic, cardiac, endocrine, or neurological conditions) and of a healthy weight (mean weight: 69 kg). Participants were randomized to receive domperidone or placebo on the first or second session by a researcher not involved in the study, using the *randi* function in MATLAB. This study was approved by the Cambridge Psychology Research Ethics Committee (PRE.2019.052). All participants provided written informed consent.

We pre-registered 40 participants, but data collection was halted by the 2020 COVID-19 pandemic. Because this is likely to fundamentally change public perception of disgust and bodily materials, we opted to stop and analyze pre-pandemic data.

### Method Details

#### General Procedure

In a randomized, placebo-controlled, double-blind, crossover design, we tested the effect of domperidone (10 mg tablet administered orally) and placebo (indistinguishable vitamin D tablet) on disgust avoidance. Participants attended two sessions at least seven days apart. As above, participants were randomized to receive domperidone or placebo on the first or second session by a researcher not involved in the study. At the first session, a physician screened each participant for medical contraindications. For the 6 hours preceding each testing session, participants were required to fast, and to avoid caffeine and other drugs, including nicotine. This allowed us to replicate the gastric conditions of previous studies where core disgust experience (visual stimuli of faeces, vomit, and pus; sham or imagined consumption of unappetising foods) elicited deviations from normogastria.^12^^,^^13^^,^^32^ At each session, following baseline task administration, participants received either domperidone or placebo in a sealed envelope. After 30 minutes (the average time to peak concentration domperidone if administered on an empty stomach), participants completed the post-administration tasks (see Figure 1). Participants did not consume clear liquids in the 45 minutes before the baseline assessment. Each participant was given 200 mL of water to consume with administration of domperidone. Post-domperidone assessments all occurred at least 30 minutes after water consumption; note that physiological responses to water consumption return to baseline levels after 30 minutes.^33^^,^^34^ Participants were compensated for their time (£9/hour), plus any winnings from the tasks.

#### Preferential looking paradigm

We employed a 24-trial preferential looking paradigm (7,8; Expt. 1), measuring gaze dwell time for disgusting versus neutral images using eye-tracking equipment at baseline. All disgusting images were of faeces, because faeces are considered the most reliable disgust elicitor.^35^ Neutral images were of household objects: a scarf, or buttons, selected to match the visual complexity of the disgusting images they were paired with.^7^ Images (resized to 400 × 300 pixels) were presented over a black background (with image centers located 640 pixels apart horizontally). The low-level visual salience of the display was previously computed and found to be well-matched between the two images.^7^ We repeated this paradigm 30 minutes post-drug-administration. We then incentivized disgust exposure through a gaze-contingent reward procedure during which participants received £0.25 (signaled by “ka-ching” sound) every 4-8 s when fixating on the disgusting image, preceded and followed by 10 non-rewarded trials (8; Expt. 3). The key outcome measure was oculomotor disgust avoidance: the difference in proportions of dwell time for neutral and disgusting stimuli. We also measured self-reported disgust ratings (see Figure 1).

#### Control paradigm (reward learning)

On each testing day, before and after domperidone or placebo administration, we also measured reward learning using a version of a robust and widely-employed instrumental conditioning paradigm. In each of the task’s 88 trials, participants chose one of two visual stimuli, indicating the left or right stimulus with the left or right arrow keys on a standard keyboard. Each stimulus was probabilistically associated with winning either £0.10 or nothing. Participants were instructed to maximize their gains during the task, which required them to learn which choices were associated with the highest probability of reward. The probability of reward shifted between an 80 and 20 percent chance of winning for each stimulus four times during the task (roughly every 20 trials) to encourage frequent updating of choices. This task is designed to measure reward processing, which is altered by central dopaminergic modulation: on a comparable task, the D2/D3 receptor antagonist sulpiride evoked profound impairments in choice performance for reward-associated stimuli.^15^ The key outcome measures were proportion of won trials, and learning rate and choice temperature parameters (parameters derived from a basic reinforcement learning model^36^). Because it has been argued that dopamine is involved specifically in learning from positive outcomes^37^, we also employed a model that yielded separate learning rate parameters for win and non-win outcomes:

(Equation 1) $$v_{A,i}=v_{A,i-1}+\eta \left(R_{i-1}-v_{A,i-1}\right)$$

(Equation 2) $$v_{A,i}=\left\{ \begin{array}{cc} v_{A,i-1}+\eta_{win}\left(R_{i-1}-v_{A,i-1}\right), & ifR_{i-1}>0\\ v_{A,i-1}+\eta_{lose}\left(R_{i-1}-v_{A,i-1}\right), & otherwise \end{array}\right.$$

With the softmax calculated as:

(Equation 3) $$P{\left(A\right)}_{i}=\frac{exp\left(\beta v_{A,i}\right)}{exp\left(\beta v_{A,i}\right)+exp\left(\beta v_{B,i}\right)}$$

Where:

• v_A,i_ is the associative value for stimulus *A* at trial *i*
• R_i_ is the reward (1 for a win, or 0 for a no-win) at trial *i*
• P(A)_i_ is the probability of choosing stimulus *A* on trial *i*
• η is the learning rate
• η_win_ is the learning rate for wins
• η_lose_ is the learning rate for non-wins
• β is the inverse choice temperatue
• Starting values for v_A_ and v_B_ are 0, ensuring P(A)_1_ = P(B)_1_ = 0.5

#### Preregistration and deviations

Our pre-registration (https://osf.io/nueps) was written to mirror^7^, including preregistered power analyses. However, during review, our mixture of repeated-measures ANOVAs and regression was suggested to be changed to LMMs, which we therefore employed here. Nevertheless, the preregistered mixed ANOVA confirms our results, accounting for sex and weight (affecting dose) as covariates (LMMs allow for this with participant-specific intercepts, as we employ). There was a drug-by-phase interaction [*F*(1,17) = 11.12, p = 0.004]; a phase-by-trial interaction [*F*(9,153) = 2.94, p = 0.003] and a drug-by-phase-by-trial interaction [*F*(9,153) = 2.64, p = 0.007]). After including baseline oculomotor disgust avoidance as covariates to control for the reported baseline offsets, the drug-by-phase interaction [*F*(1,15) = 9.33, p = 0.008], phase-by-trial interaction [*F*(9,135) = 3.61, p < 0.001], and drug-by-phase-by-trial interaction [*F*(9,135) = 2.59, p = 0.009] all remained statistically significant.

#### Sample size estimation

In addition to our preregistered *a priori* power analyses, due to the deviation in sample size, we conducted post-data-collection power analyses to inform our analytical approach. At 80% power, our reduced sample size would only detect correlations of *r* > 0.53, so we did not perform any planned correlations between gaze and self-report outcomes. In contrast, a repeated-measures ANOVA to detect a medium effect size (*f =* 0.3) requires at least 24 participants to achieve 80% power (calculated in G^∗^Power 3.1: *F-*tests; ANOVA: repeated-measures, within factors; alpha = 0.05). Therefore, our reduced sample size of N = 27 would have been sufficient to detect at least medium-sized effects with our preregistered analysis approach.

### Quantification and Statistical Analysis

For analysis of the preferential looking paradigm, linear mixed models (LMMs) were fit to trial-level dwell time proportions, with participant number as a random effect (thereby implicitly accounting for inter-individual differences in sex and weight), and factors drug (domperidone/placebo), stimulus (disgust/neutral), phase (baseline/post-administration or pre-/post-exposure), and trial number; and their interactions. Trials with > 50% missing data were dropped (preferential looking: 26/2496, encouraged exposure: 14/1560). For self-reported disgust, trial number was not a factor.

The reward learning control task was analyzed in the same way, using LMMs with factors drug, phase, and their interaction. These were fitted to each outcome measure (summarized above) independently. In addition, to make credible any claims of a null effect, we compared these LMMs to null (intercept-only) models. Furthermore, we employed post hoc LMMs testing only the effect of drug on the difference in outcome measure between the placebo and domperidone, and again compared these to null models.

The absence of a drug effect could be supported by three pieces of evidence. The first was a lack of a significant main effect of drug in a post hoc test (*p* > 0.05), which can indicate the lack of an effect, but is not direct evidence of it. More direct evidence comes from comparisons against null models, and was quantified in two ways: as the Evidence Ratio (Equations 4 and 5) computed from the Akaike Information Criterions (AIC) for a model and associated null model^38^, and as Bayes Factor (Equation 6) computed without the need for priors from the Bayesian Information Criterions (BIC) for a model and its associated null model.^39^

Resulting Evidence Ratios and Bayes Factors in favor of null hypotheses (ER_0_ and BF_01_) quantify how much more likely the null hypothesis is, compared to the alternative. They can also be interpreted according to Jeffreys’ guidelines^40^, with values over 3 as evidence for the null. Therefore, in the Results, we report all three of these statistical approaches: from the LMMs, beta values representing effects/interaction effects, their confidence intervals, *Z* values, and *p* values; from the AIC and BIC, ER_0_ and BF_01_ respectively.

(Equation 4) $$w_{i}=\frac{exp\left(-0.5\left(AIC_{i}-AIC_{min}\right)\right)}{exp\left(-0.5\left(AIC_{0}-AIC_{min}\right)\right)+exp\left(-0.5\left(AIC_{1}-AIC_{min}\right)\right)}$$

(Equation 5) $$ER_{0}=w_{0}/w_{1}$$

(Equation 6) $$BF_{01}=e^{\left(0.5\left(BIC_{1}-BIC_{0}\right)\right)}$$

### Additional Resources

Preregistration: https://osf.io/nueps
